# Numerical evaluation of spinal reconstruction using a 3D printed vertebral body replacement implant: effects of material anisotropy

**DOI:** 10.3389/fbioe.2024.1305837

**Published:** 2024-06-20

**Authors:** Jianfeng Kang, Yanlong Wu, Jian Qiao

**Affiliations:** School of Mechatronic Engineering and Automation, Foshan University, Foshan, China

**Keywords:** spinal reconstruction, biomechanical properties, finite element analysis, linear elastic isotropy, nonlinear anisotropy

## Abstract

**Background and objective:**

Artificial vertebral implants have been widely used for functional reconstruction of vertebral defects caused by tumors or trauma. However, the evaluation of their biomechanical properties often neglects the influence of material anisotropy derived from the host bone and implant’s microstructures. Hence, this study aims to investigate the effect of material anisotropy on the safety and stability of vertebral reconstruction.

**Material and methods:**

Two finite element models were developed to reflect the difference of material properties between linear elastic isotropy and nonlinear anisotropy. Their biomechanical evaluation was carried out under different load conditions including flexion, extension, lateral bending and axial rotation. These performances of two models with respect to safety and stability were analyzed and compared quantitatively based on the predicted von Mises stress, displacement and effective strain.

**Results:**

The maximum von Mises stress of each component in both models was lower than the yield strength of respective material, while the predicted results of nonlinear anisotropic model were generally below to those of the linear elastic isotropic model. Furthermore, the maximum von Mises stress of natural vertebra and reconstructed system was decreased by 2–37 MPa and 20–61 MPa, respectively. The maximum reductions for the translation displacement of the artificial vertebral body implant and motion range of whole model were reached to 0.26 mm and 0.77°. The percentage of effective strain elements on the superior and inferior endplates adjacent to implant was diminished by up to 19.7% and 23.1%, respectively.

**Conclusion:**

After comprehensive comparison, these results indicated that the finite element model with the assumption of linear elastic isotropy may underestimate the safety of the reconstruction system, while misdiagnose higher stability by overestimating the range of motion and bone growth capability.

## 1 Introduction

Spinal column, regarded as the central axis of human skeleton system, possesses multiple functions of load-bearing, shock absorption, protection, and movement. Unfortunately, traumatic injury, congenital defects or surgical removal of tumors can result in large defects or absences of vertebrae that require clinical intervention if functional restoration is to be achieved. Currently, this combination of artificial vertebral body implants and different fixation strategies is usually used to accomplish vertebral replacement and functional reconstruction (Zhang and Guo, 2023). Moreover, some 3D printed patient-specific implants considering fully the differences of individualized defects and anatomical morphology, are also designed and applied to harvest better service performance in clinic (Hu et al., 2022; Palmquist et al., 2023). However, several postoperative complications still existed (Yoshioka et al., 2013), such as subsidence of implants, screw misplacement and pedicle breakage, degeneration of adjacent segments, etc. These risks are closely associated with the biomechanical properties of the spinal reconstruction system, and portend that further investigation for influence factors of biomechanical performance will provide a solid foundation for improving clinical outcomes.

The Wolff’s law pointed out that host bones can adapt to satisfy functional response demands by changing their internal structure and external morphology. Under the action of complex physiological loads and motions, the hierarchical and porous structure of natural bone exhibits mechanical anisotropy, further providing excellent stress distribution and effectively maintaining a dynamic balance between bone formation and resorption. In this scenario, the anisotropic behaviour of mechanical properties for the cortical or cancellous bone in various vertebrae was investigated by using micro computed tomography (μCT) scanning analysis (Perilli et al., 2012), micro-scale finite element method (Goda and Ganghoffer, 2015), uniaxial loading tests (Yeni et al., 2022), nanoindentation test (Wolfram et al., 2010) and ultrasound measurements (Nicholson and Alkalay, 2007). These studies fully confirmed the significant anisotropy of mechanical properties for the host bone, such as the anisotropic degree of thoracic and lumbar vertebral segment reached to 1.47 ± 0.17 and 1.51 ± 0.21, respectively (Lochmüller et al., 2008). Great variability of mechanical properties was closely related to the anatomic location, orientation, and nonhomogeneous morphology (Hulme et al., 2007). However, the material properties of natural vertebrae in the process of biomechanical analysis were set as the linear elastic isotropy (Fan et al., 2021), or based on the mapping relationship of the grayscale-apparent density-modulus (Gong et al., 2022). Hence, it is indispensable and urgent to take full account of the influence of material anisotropy during the design and biomechanical evaluation of bone defect repair, so as to accurately reflect the service performance of the reconstructed system.

Artificial vertebral body implants constructed with a combination of solid and porous structures, can obtain integrated advantages of light weight, high strength and superior stability through the rational design of macro/microstructures and additive manufacturing technology (Kang et al., 2021), such as the 3D ACT vertebral body prosthesis from Beijing AKEC Medical Co., Ltd., the F3D corpectomy vertebral body replacement system from CoreLink LLC., etc. These certified implants were prepared using a variety of porous lattice types, controllable geometric parameters, as well as different powder bed fusion 3D printing technologies including selective laser melting and electron beam melting. Overall, the mechanical properties of porous lattices and their influencing factors have been extensively studied based on the homogenization theory, finite element analysis and experimental measurements, further better serving the design and modeling of 3D printed medical implants (Wang et al., 2017). Nevertheless, the anisotropy of mechanical properties for the implant’s microstructure was notable and have been studied (Barba et al., 2019; Jia et al., 2023). In our previous study (Kang et al., 2020), the numerical method for modulus anisotropy of porous structures was developed to characterize the spatial distribution of elastic modulus and degree of anisotropy effectively. Despite multiple benefits attributed to the porosity design of medical implants, the anisotropic mechanical properties of porous structures should also be paid more attention and analyzed accurately in the evaluation of biomechanical performance.

The finite element method was widely used to explore the biomechanical performance of vertebral defect repair under various loading and activities conditions similar with *in vivo* environment (Dong et al., 2020; Dai et al., 2022; Mehboob, 2023). After the numerical calculation, the safety and stability of spinal reconstruction system can be evaluated quantitatively. However, the material properties of cancellous or cortical bone in the finite element analysis were frequently simplified as linear elastic isotropy (Dong et al., 2020; Kang et al., 2021). Directional dependent variation in mechanical properties for the implant’s microstructure have not been fully considered (Wang et al., 2016). Overall, existing finite element models for the biomechanical analysis of vertebral reconstruction systems rarely take into account the effect of material anisotropy from host bone and implant’s microstructure on their biomechanical properties, which may cause significant discrepancies between real service performance and design expectations.

Oriented to the clinical complications of vertebral body replacement, the study aims to understand the effect of material anisotropy of component materials on the biomechanical properties of spinal reconstruction. Hence, two finite element models considering anisotropic difference were developed to evaluate and compare the biomechanical properties under various loads conditions. This study not only reveals the influence of different material properties on biomechanical properties, but also provides an effective methodology for the design and performance evaluation of artificial vertebral implants.

## 2 Materials and methods

### 2.1 Reconstruction of geometrical models

**FIGURE 1:**
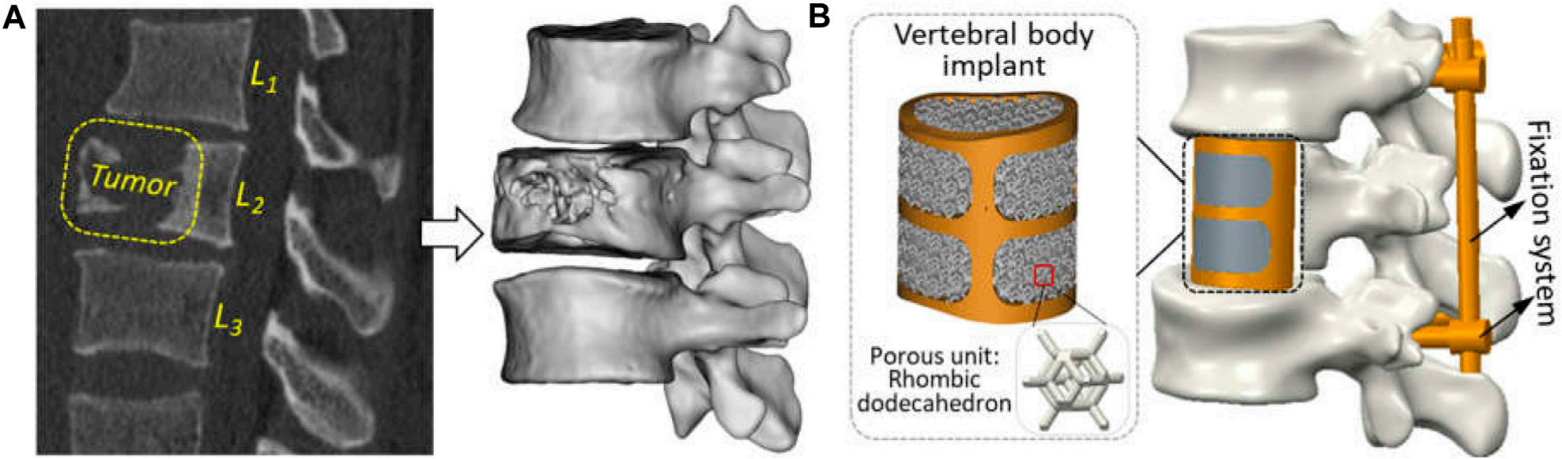
Reconstruction of geometric models for clinical treatment of vertebral defect: **(A)** tumor observation in the CT scanning image and three-dimensional geometric modeling; **(B)** vertebral replacement using by the artificial vertebral body implant and posterior fixation system.

According to the CT images of a 25-year-old male patient provided by Xijing Hospital (the First Affiliated Hospital of the Fourth Military Medical University, Shaanxi, China), malignant tumor location can be clearly observed in the second segment of the lumbar vertebra, accompanied by severe vertebral erosion symptoms, as shown in Figure 1A. Therein, the exported DICOM files with a slice thickness of 0.625 mm and a pixel size of 0.35 mm ensured the reconstruction of three-dimension geometrical models for the spine and tumor. Briefly, the modeling process was to import CT images into the Mimics software (Version 17.0, Materialise, Inc., Leuven, Belgium), extracting the three-dimensional geometric model of target components separately by setting different gray thresholds, and then import the outputted STL files into the Geomagic Wrap software (Version 2017, 3D System, Inc., United States) for the operation of polygon smoothing and exact surfacing. Based on the patient’s actual situation, the preoperative planning was determined to be complete resection of the tumor, attached locally to the vertebral segment and adjacent intervertebral discs. And then the repair of vertebral defect was designed with artificial vertebral body implant combining with posterior fixation system.

Under the guidance of clinical surgeons, numerical simulation of tumor resection was performed to measure some key characteristic parameters of the defect region, such as the height, transverse diameter, sagittal diameter, etc. Based on the aforementioned parameters, a patient-specific artificial vertebral body implant with solid beams and porous structure was designed. Since the design of vertebral body implant was investigated in previous studies (Shi et al., 2020), a traditional trussed vertebral implant with solid wall thickness of 2 mm was used directly. Its macro geometric shape adopts the orthogonal combination of transverse and longitudinal beam in the anterior and solid thin wall in the posterior. Based on existing studies (Shi et al., 2020; Kang et al., 2021), a rhombic dodecahedron unit with a length of 2 mm, a porosity of 80%, and a strut diameter of 0.3 mm possessed excellent bone-ingrowth capability and has been applied clinically. So, this porous lattice was used to fill inside vertebral implant. Additionally, the posterior fixation system, including pedicle screws (*ϕ*×*L*: 4 × 40 mm) and connecting rods (*ϕ*×*L*: 5 × 75 mm), were fabricated by traditional machining methods using Ti_6_Al_4_V material and used to further enhance the stability of reconstructed model.

### 2.2 Finite element modeling

Based on the above geometric model, the responding finite element model was developed in the Abaqus software (Version 6.14, Dassault Systems Inc., France). To evaluate the influence of material anisotropy on the biomechanical properties of vertebral body replacement, different material properties with linear elastic isotropic and nonlinear anisotropic features for each component of the reconstructed lumbar spine model were assigned as given in Table 1. The effective elastic modulus and yield strength of porous structure was measured by compression (Dong et al., 2020; Kang et al., 2021). For another, the modulus anisotropy of porous structures considering the irregular geometry after 3D-printing was analyzed by the numerical method developed of previous study (Kang et al., 2020). Besides, corresponding ligaments were integrated into the model through nonlinear springs to define the tension-only and incompressible behaviours (Kang et al., 2021), as shown in Table 2.

**TABLE 1:** Assignment of materials properties for each component.

Models	Components	Properties parameters	References
**Linear elastic isotropic model**	Cortical bone	*E* = 12 GPa; *v* = 0.3	Lu et al. (2022)
	Cancellous bone	*E* = 100 MPa; *v* = 0.2	Lu et al. (2022)
	Solid part of vertebral body implant	*E* = 110 GPa; *v* = 0.3	Dong et al. (2020), Kang et al. (2021)
	Porous part of vertebral body implant	*E* = 800 MPa; *v* = 0.3	Dong et al. (2020), Kang et al. (2021)
	Fixation system with Ti_6_Al_4_V material	*E* = 110 GPa; *v* = 0.3	Dong et al. (2020), Kang et al. (2021)
**Nonlinear anisotropic model**	Cortical bone	*C*_11_ = 11.13, *C*_22_ = 11.13, *C*_33_ = 15.37 *C*_44_ = 6.92, *C*_55_ = 6.92, *C*_66_ = 5.89, *C*_12_ = 5.24, *C*_23_ = *C*_13_ = 6.15	Wolfram et al. (2010)
	Cancellous bone	*C*_11_ = 152, *C*_22_ = 152, *C*_33_ = 539, *C*_44_ = 1.8, *C*_55_ = 3.2, *C*_66_ = 3.2, *C*_12_ = 4.0, *C*_23_ = *C*_13_ = 11.0	Goda and Ganghoffer (2015)
	Porous part of vertebral body implant	*C*_11_ = *C*_22_ = *C*_33_ = 2.405, *C*_44_ = 1.705, *C*_55_ = 1.845, *C*_66_ = 3.538, *C*_12_ = *C*_23_ = *C*_13_ = 1.875	Kang et al. (2020)

**TABLE 2:** Materials properties of different ligaments in the finite element model.

Materials	Young’s modulus (MPa)	Sectional area (mm^2^)	Stiffness (N/mm)
Transverse ligament	10	1.8	0.9
Interspinous ligament	10	70	35
Ligamentum flavum	15	40	30
Capsular ligament	7.5	30	10
Supraspinous ligament	10	70	35
Posterior longitudinal ligament	10	20	10

**FIGURE 2:**
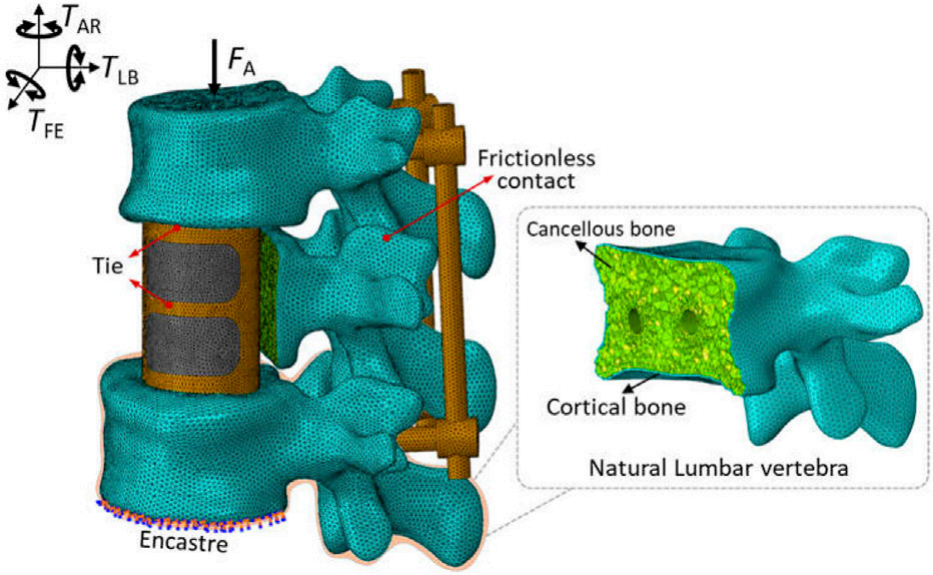
The basic setups of finite element model of artificial vertebral body replacement.

Each component was meshed using the Hypermesh software (Version 12.0, Altair Engineering, Inc., United States) and then imported into Abaqus software for a finite element analysis, as shown in Figure 2. According to existing studies (Kang et al., 2021) combined with the statistical measurements of the patient’s CT image data, the triangular prism element (C3D6) with a layer of 0.4 mm thick was used to reflect the role of cortical bone. The C3D6 element can ensure good stress transfer at the interface between cortical bone and cancellous bone through the co-node feature. The cancellous bone and the remaining components were meshed using a tetrahedron element (C3D4). To remove the impacts of meshing size, a meshing sensitivity analysis was carried out under the conditions of meshing sizes of 0.5 mm, 1 mm and 2 mm. Less than a 5% relative difference was achieved across all sizes, thus 1 mm was selected. Overall, 782,804 elements were included for a complete model.

A “tie” constraint was set for the contact interface between the endplate and vertebral body implant, as well as between the pedicle screws and bone. Facet joint articulation at all levels was simulated as a finite sliding contact problem with frictionless property owing to the cartilaginous effect. These settings of contact mode were widely accepted in the studies (Dreischarf et al., 2014; Tsouknidas et al., 2015). Additionally, the boundary and loading conditions were shown in Figure 2. For the spinal finite element model, it is very common to fix the lower surface of the bottom vertebrae and apply load to the upper surface of the top vertebrae to simulate the human spinal daily activities (Cao et al., 2001; Vadapalli et al., 2006; Dreischarf et al., 2014). The inferior endplate of vertebra *L*_3_ was fully constrained, and a vertical load of 400 N and a moment of 10 Nm from different directions were applied on the upper surface of the *L*_1_ segment to simulate flexion, extension, lateral bending and axial rotation according to normal vertebral activity (Wang et al., 2018).

### 2.3 Biomechanical evaluation

After finishing the numerical calculation, the von Mises stress, displacement and effective strain of each component were extracted to analyze the safety and stability of spinal reconstruction. The safety evaluation was carried out by comparing the maximum von Mises stress of each component with the yield strength of its own material. The initial stability of the reconstruction system was assessed by the translational and rotational displacement, and medium-long term stability by the effective strain to stimulate bone ingrowth. According to the bone’s mechanostat (Frost, 2003), the effective strain range for maintaining bone balance and bone remodeling is 200–5,000 *με*, with the minimum effective strain threshold for remodeling being 200 *με*, modeling being 1,000 *με*, and pathologic microdamage being 3,000-5,000 *με*. Hence, the effective strain on the vertebral endplate adjacent to the implant was employed to reflect the potential of bone remodeling.

## 3 Results

### 3.1 Safety evaluation

**FIGURE 3:**
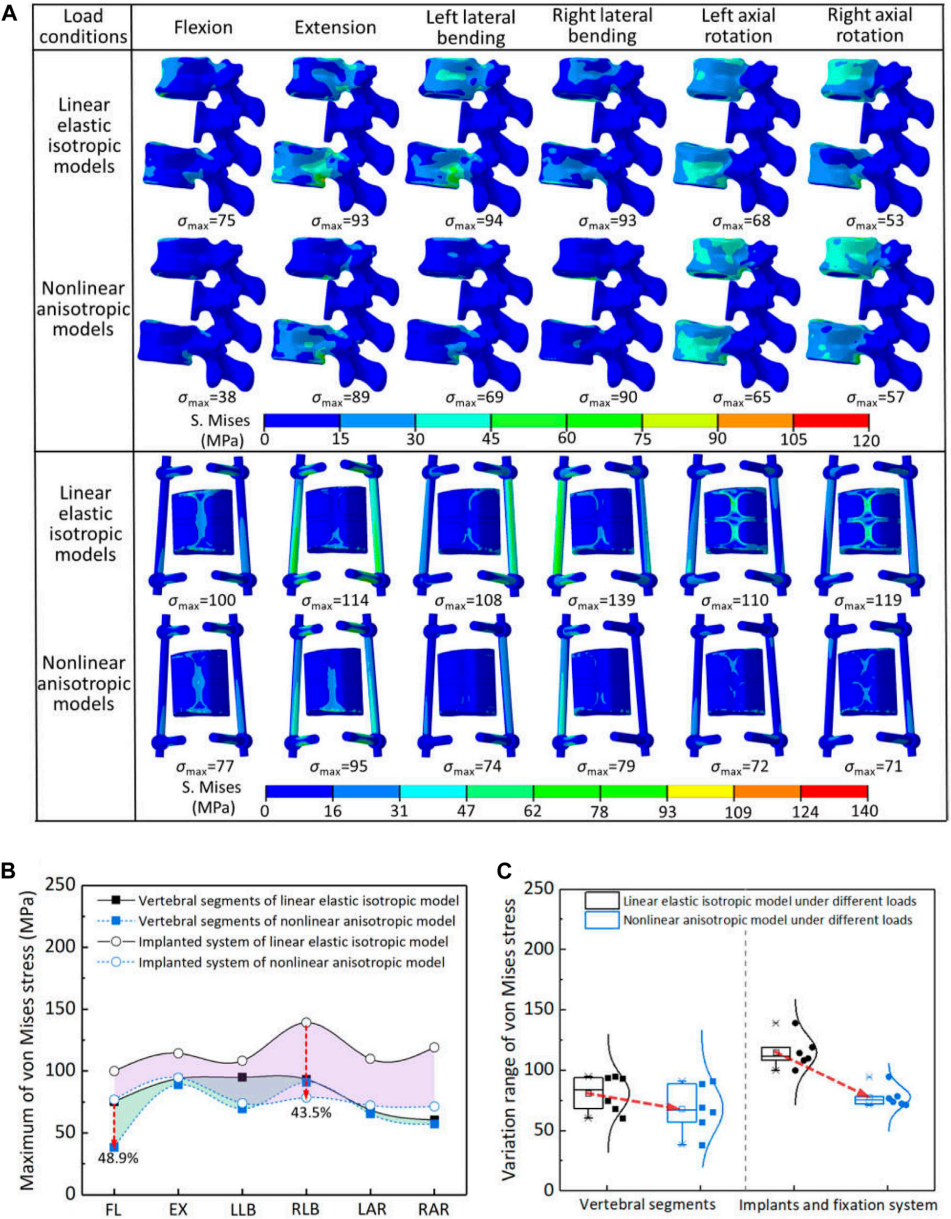
The von Mises stress results of two models under different load conditions: **(A)** the stress distribution of each component; **(B)** comparison of stress reduction between natural vertebra and reconstructed system (FL—flexion, EX—extension, LLB—left lateral bending, RLB—right lateral bending, LAR—left axial rotation, RAR—right axial rotation); **(C)** statistical analysis for the variation range of von Mises stress.

The stress distribution and statistical analysis results of each component of the spinal reconstruction model under different load conditions were shown in Figure 3. For the linear elastic isotropic models, the variation range of the maximum von Mises stress for the natural vertebrae and reconstruction system (including the artificial vertebral implant and posterior fixation system) under all load conditions was 75–94 MPa and 100–139 MPa, respectively. Among them, the maximum von Mises stress of natural vertebrae during lateral bending and extension was higher than that of the remaining load, which was mainly located at the contact interface between the vertebral implant and adjacent endplates. The maximum von Mises stress of the reconstruction system was the highest under the right axial rotation, and it could be seen from the stress distribution that the truss structure of the artificial vertebral implant played an important load-bearing role. For the nonlinear anisotropic model, the maximum von Mises stress of natural vertebra and reconstructed system under all loading conditions was lower than that of the linear elastic isotropic model, which were 38–90 MPa and 71–95 MPa, respectively. The maximum von Mises stress of the natural vertebra was the highest in the right lateral bending and extension movements, while the reconstruction system was the highest in the extension movement. On the whole, taking the anisotropic properties of the host bone and microstructure of vertebral implant into account, the loading mode for generating the maximum von Mises stress was changed, and the maximum von Mises stress can be effectively reduced.

The maximum von Mises stress of natural vertebrae and reconstructed system were further quantitatively compared in the two models, as shown in Figure 3B,C. When considering anisotropy, the decrease percentage of the maximum von Mises stress for the natural vertebrae and reconstructed system under all loading conditions was 2.6%–48.9% and 17.3%–43.5%, respectively. The natural vertebrae were the most reduced in the flexion motion, while the reconstructed system was the right lateral bending. Overall, the maximum von Mises stress of all components in the two models was less than the yield strength of the materials. Therefore, according to the results of von Mises stress, it can be seen that the linear elastic isotropy setting may underestimate the safety of the reconstruction system. Namely, the safety of spinal reconstruction will be safer than the assumption of linear elastic isotropy.

### 3.2 Displacement of movement

**FIGURE 4:**
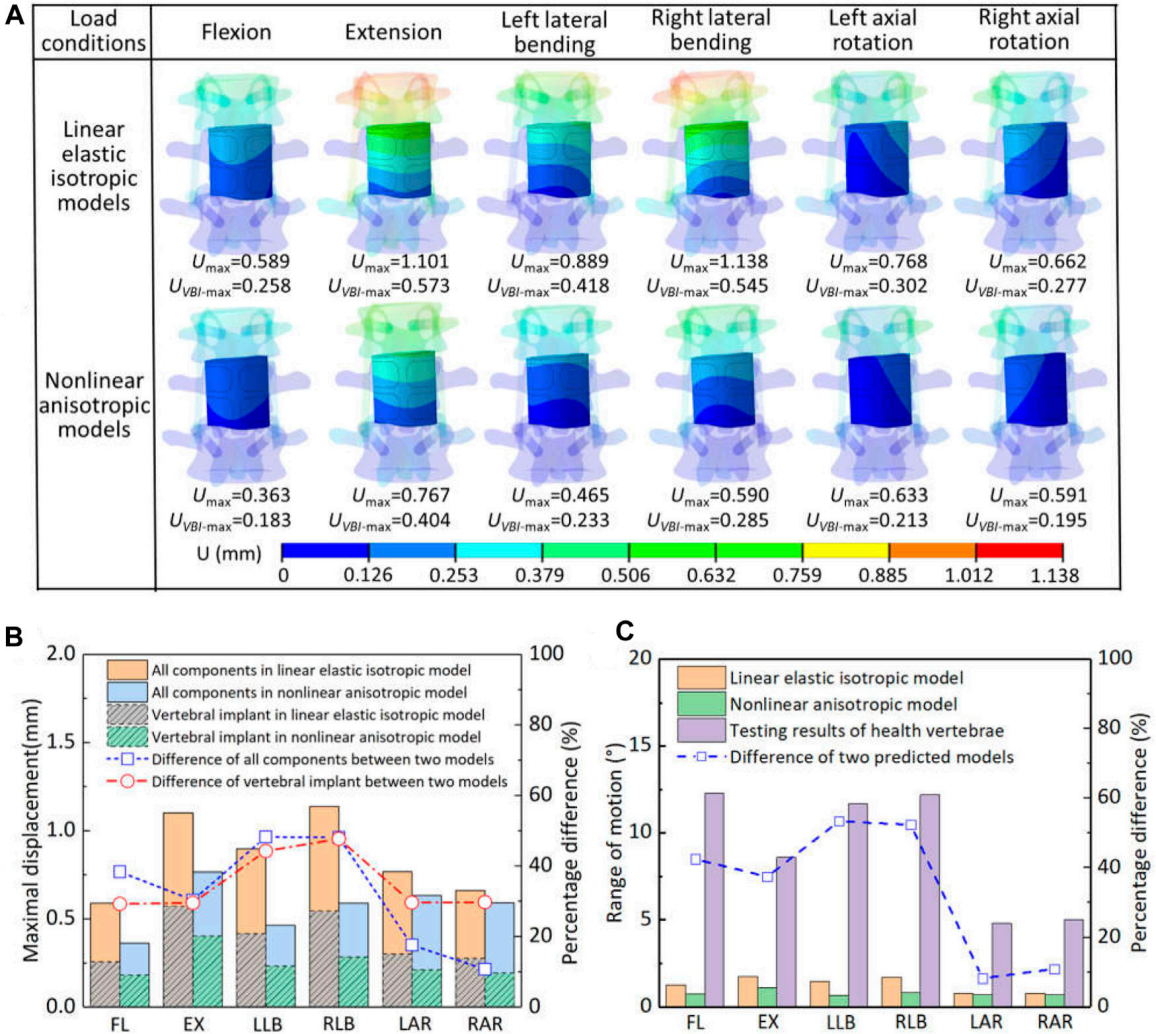
The displacement and range of motion for the two models under different load conditions: **(A)** the displacement distribution of artificial implant; **(B)** comparison of maximal displacement of all components and artificial implant in the two models; **(C)** comparison of the range of motion.

The displacement distribution and range of motion for the spinal reconstruction model under different load conditions were shown in Figure 4. Overall, the displacement distribution trend of each component in the two models was similar, while the maximum displacement was different. For the linear elastic isotropic model, the maximum displacement under the right lateral bending was relatively larger than that of other motions, but the corresponding law was found in the extension for the nonlinear anisotropic model. In addition, the maximum displacements of all components and artificial vertebral implant in the two models were further analyzed, and it was found that the calculation results of the nonlinear anisotropic model were lower than those of the linear elastic isotropic model in the left and right lateral bending motion, with a reduction range of 44.3%–48.3%. The maximum displacement of artificial vertebral implant can account for half of the those of the whole model in flexion, extension and lateral bending motion, but the proportion decreases to 32.9%–41.8% for axial rotation motion. In addition, compared with the linear elastic isotropic model, the range of motion for the nonlinear anisotropic model decreased in the range of 8.2%–53.4%. With reference to the mobility of the normal human vertebrae (*L*_1_—*L*_3_), there was a significant reduction in overall mobility due to strong posterior fixation system, with an overall reduction of essentially 79.5%–94.2% in both models. Overall, from the results of displacement and range of motion, the artificial vertebral body implant has better initial stability when considering the anisotropic properties.

### 3.3 Bone remodeling capability

**FIGURE 5:**
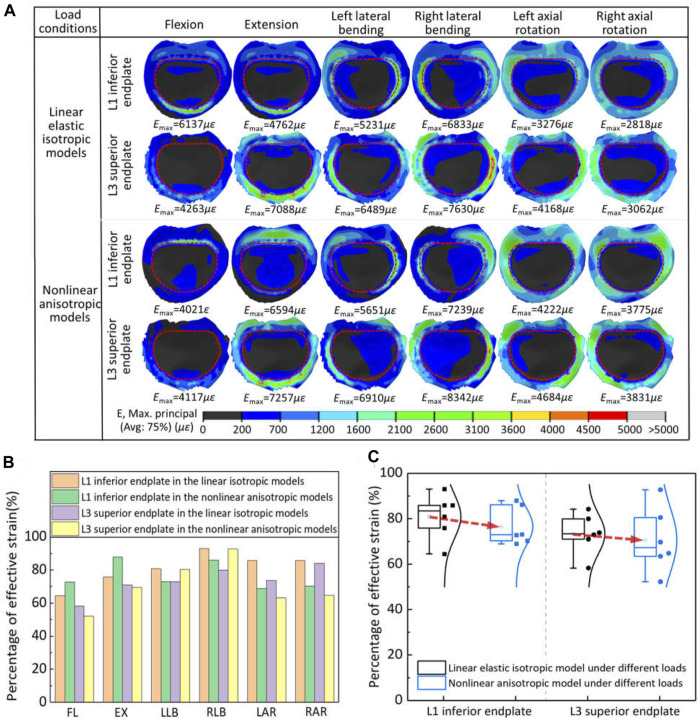
The effective strain results on the vertebral endplates adjacent to the artificial implant in two models: **(A)** the effective strain distribution of vertebral endplates; **(B)** comparison of percentage of effective strain in two models under different loads; **(C)** statistical analysis for the variation range of effective strain in the two models.

The effective strain distribution for bone growth stimulation and its statistical analysis on the vertebral endplates adjacent to the artificial implant in two models were shown in Figure 5. The maximum strain and distribution of vertebral endplate were different under different load conditions due to different settings of material properties. Except for the flexion and extension, the maximum strain values for the inferior and superior vertebral endplates of the linearly elastic isotropic model were lower than those of the nonlinearly anisotropic model under all motion conditions, with increases ranging from 2.4% to 38.5%, and the maximum strain values occurred at roughly similar locations. But the flexion-extension motion showed the opposite tendency, with decreases of 34.5% and 3.4% respectively. Moreover, the maximum strain of *L*_1_ inferior endplate changed from the anterior region to the posterior end. In addition, more strain values for maintaining bone balance were observed inside the contact surface between the vertebral endplate and the artificial vertebral implant, while effective strain values for stimulating bone growth were observed in the marginal area of the contact surface.

The percentage of effective strain elements on the inferior/superior vertebral endplate stimulating bone growth in the two models was further extracted for statistical analysis. For the axial rotation, the effective strains in the vertebral endplates of the linear elastic isotropic model were higher than those of the nonlinear anisotropic model, with a range of 14.1%–23.1%. For lateral bending and flexion-extension motion, the effective strains in the vertebral endplates may show a pattern of strain transfer or compensation. For example, the effective strains in the *L*_1_ inferior endplate of the linear elastic isotropic model were lower than those of the nonlinear anisotropic model under the flexion-extension movements, but this pattern was reversed for the *L*_3_ superior endplate. Finally, the proportion of strain units that effectively stimulated bone growth calculated by the linear elastic model was significantly higher than that calculated by the nonlinear anisotropic model, indicating that the setting of linear elastic isotropic material properties may overestimate the bone growth ability to some extent.

## 4 Discussion

Two finite element models for linear elastic isotropy and nonlinear anisotropy were developed to evaluate the effect of different material properties on the safety of vertebral reconstructed system. For above two models, the maximum von Mises stress of the natural vertebrae was 95 MPa and 90 MPa, respectively, and that of the reconstructed system was 139 MPa and 95 MPa. Among, the maximal stresses of these natural vertebrae were lower than the yield strength of material itself (104.9–114.3 MPa) (Haghshenas, 2017). Morover, the stress distribution of artificial vertebral body implant showed that the solid truss structure plays an important role in load-bearing aspect, and these maximal stresses under various loads were also below the reported fatigue strength of 3D printed solid samples (about 200–300 MPa) (Denti et al., 2019; Liu and Shin, 2019). Overall, the maximum von Mises stress calculated by both models for each component of the spinal reconstructive system was less than the yield strength of the material itself. However, the predicted results of the linear elastic model were higher than those of the nonlinear anisotropic model, which indicated the setting of linear elastic isotropy will overestimate the safety when compared with the anisotropic model.

**FIGURE 6:**
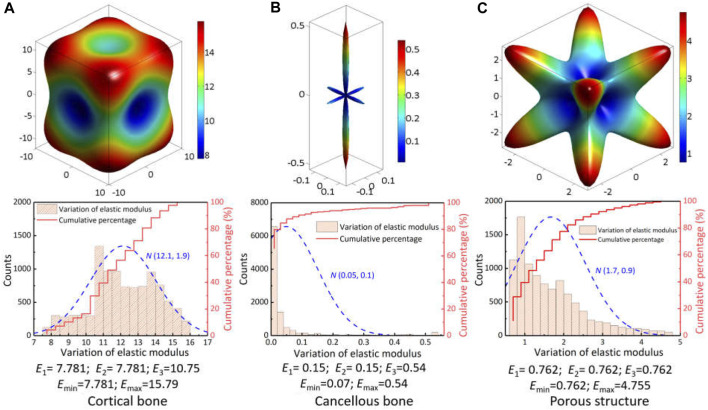
Spatial distribution of elastic modulus for the vertebral skeleton structures and implant’s porous structure: **(A)** cortical bone; **(B)** cancellous bone; **(C)** porous structure.

After comparing the predicted results of two finite element models, it can be seen that the assignment of anisotropic properties not only changed the stress distribution pattern under different load conditions, but also effectively reduced the value of maximal stress. This variation was mainly attributed to the diversity in the spatial distribution of elastic modulus between natural vertebrae and implant’s microstructure. From their anisotropic parameters in Table 1, the spatial surface of elastic modulus was plotted and variation ranges were statistically analyzed by normal distribution, as shown in Figure 6. For the cortical bone of natural vertebrae, the maximal and minimal modulus were located on the body diagonal direction and the axial direction with a variation of 7.78 GPa–15.79 GPa, and the percentages for below and above 12 GPa were roughly half. Especially for cancellous bone, the spatial distribution of elastic modulus varied significantly owing to the influence of porosity variety and sampling site. Despite large difference for the minimal and maximal value for the elastic modulus, nearly 90% was between 0.07 GPa and 0.1 GPa. In addition, for the porous structure of the artificial vertebral body implant, the spatial distribution of elastic modulus was closely related to the geometry of porous structure, which has been confirmed by previous studies (Kang et al., 2020). The rhombic dodecahedral porous structure belongs to centrosymmetric, resulting in predicted elastic modulus of 0.762 GPa in all three axial directions, which was close to the experimental measurement of 0.8 GPa (Dong et al., 2020). But the elastic modulus in the body diagonal direction was as high as 4.75 GPa and more than 75% was greater than 0.8 GPa. Hence, due to these discrepancy in spatial distribution of elastic modulus, it is necessary to fully consider and understand the influence of material anisotropy on the biomechanical properties of spinal reconstruction system.

Due to strong protection of the posterior fixation system, the maximum translational displacement of vertebral implant for two models under all load cases were 0.573 mm and 0.404 mm, respectively, which occurred in extension motion. Compared with the testing results of heath vertebrae at *L*_1_—*L*_3_ segments (Yamamoto et al., 1989), the predicted range of motion for two models decreased significantly, with a decrease of 79.5%–94.2%.In addition, some studies (Dong et al., 2020; Zhang and Guo, 2023) on reliability of different fixation strategies pointed out that posterior fixation system exhibited strong effect compared with other fixation methods. According to the spatial distribution difference of elastic modulus given in Figure 6, the response displacement generated under the actual load-bearing process was different. Overall, the predicted results of nonlinear anisotropic model were smaller than those of linear elastic model, which indicated the setting of linear elastic isotropy may overestimate the mobility of the reconstruction system.

Owing to the lack of human spine specimens, indirect validation of the finite element models was mainly performed by comparing the results of existing biomechanical experiment testing and numerical simulations. First, the finite element model of the intact lumbar segments was validated by the comparing the range of motion values from the reported experiment data in our previous study (Dong et al., 2020). These results showed that the predicted range of motion was in good agreement with the *in vitro* experimental data or numerical results. On the basis of the previous finite element model, the effect of material properties on biomechanical properties was evaluated in this study. Moreover, the predicted range of motion under the condition of flexion, extension and lateral bending (0.728 mm, 1.1 mm and 0.8 mm) was closer to the experimental results of corresponding motions (0.6 ± 0.37 mm, 1.56 ± 0.74 mm and 0.87 ± 0.55 mm) (Disch et al., 2008). However, it should be noted that the performance of artificial vertebral implants *in vivo* will be affected by a combination of factors (Dreischarf et al., 2014; Liebsch et al., 2020a; Liebsch et al., 2020b), such as geometrical morphology of different human vertebrae, the design and material of artificial vertebral implants, internal fixation systems and clinical implantation biases, etc. According to the single factor rule, this study can also effectively evaluate the influence of different material property on biomechanical performance to a certain extent.

Comparing further the calculation results of two models under the lateral bending and axial rotation in the left and right direction, it can be shown that the maximal von Mises stress (108 MPa) and maximal displacement (0.889 mm) under the left lateral bending were lower than that of the right lateral bending (139 MPa and 1.138 mm). Yet, both of the above results under the left and right axial rotation exhibited an opposite rule, namely, maximal von Mises stress located in the left axial rotation, maximal displacement within the right axial rotation. These differences have also been observed in existing studies (Dai et al., 2022; Hao et al., 2023), and were resulted from multiple factors, such as the asymmetry of natural vertebrae model in the sagittal plane, actual placement of artificial vertebral implant and posterior fixation system, the consideration of anisotropic materials properties, etc. Consequently, this also indicated it is necessary to take into account the loading effects along the left and right direction when the biomechanical evaluation of vertebral reconstruction.

It was found that the linear elastic model had a higher percentage than the anisotropic model with respect to the element units to stimulate bone ingrowth and maintain bone balance. Moreover, the consideration of anisotropic mechanical properties brought a regional transfer or compensation of the effective strain in the vertebral endplate adjacent to the artificial implant. These variations were mainly caused by the anisotropy of elastic modulus for different components. According to the spatial distribution and statistical analysis for the elastic modulus of porous structure in Figure 6C, it can be seen that the elastic modulus in most directions was greater than 0.8 GPa set in the linear elastic isotropic model, which resulted in the increasement of maximum strain as shown in Figure 5A. The response trend was consistent with existing study (Lu et al., 2022), in which the relationship between the elastic modulus of cage materials (0.1 GPa–110 GPa) and the biomechanical properties of transforaminal lumbar interbody fusion was developed. These differences of effective strain result implied that the setting of linear elastic isotropy may overestimate the bone remodeling potential to some extent.

Some limitations of this work must also be noted. The simplification treatment by using solid elements with equivalent mechanical properties instead of porous structures was widely applied in the finite element analysis. Nevertheless, many geometrical models of artificial vertebral body implants constructed by employing Boolean operations tend to form incomplete porous units on their contoured surfaces. So, this treatment still needs to be explored further due to the scale effect and discrepancies in the mechanical properties of various incomplete porous units. In addition, the anisotropic property of this study mainly focused on the elastic deformation stage, actually the yield strength with directional dependence. Despite the choice of minimal yield strength in different directions to ensure overall safety, subsequent study also was carried out by introducing the anisotropic strength to develop more accurate evaluation method. Finally, since this study focused on the influence of anisotropy with finite element methods, there are still some problems that should be given sufficient attention, such as the asymmetry of geometric model, validation of material properties, lack of experimental testing to verify the analysis results.

## 5 Conclusion

In this study, the influence of material anisotropy derived from the skeleton structure of natural vertebrae and implant’s microstructure on the biomechanical properties of spinal reconstruction system was investigated under various load conditions. The safety, initial and long-term stability for the spinal reconstruction system with linear elastic isotropy and nonlinear anisotropy were compared to better guide clinical repair. Through finite element analysis, the predicted results of nonlinear anisotropic model showed smaller maximum von Mises stress, lower translational and rotational displacement, less element percentage of effective strain compared with the linear elastic isotropic model. It can be concluded that the assumption of linear elastic isotropy in the biomechanical evaluation of spinal reconstruction system may underestimate the safety, while overestimating the initial and long-term stability as reflected by the displacement of movement and bone ingrowth capacity.

## Data Availability

The raw data supporting the conclusion of this article will be made available by the authors, without undue reservation.
